# A non-contrast computed tomography-based radiomics nomogram for the prediction of hematoma expansion in patients with deep ganglionic intracerebral hemorrhage

**DOI:** 10.3389/fneur.2022.974183

**Published:** 2022-10-11

**Authors:** Wei Xu, Hongquan Guo, Huiping Li, Qiliang Dai, Kangping Song, Fangyi Li, Junjie Zhou, Jingjiang Yao, Zhen Wang, Xinfeng Liu

**Affiliations:** ^1^Department of Neurology, Jinling Hospital, The First School of Clinical Medicine, Southern Medical University, Nanjing, China; ^2^Department of Neurology, The Affiliated Changsha Central Hospital, Hengyang Medical School, University of South China, Changsha, China; ^3^Department of Rehabilitation, Hunan Provincial People's Hospital (The First-Affiliated Hospital of Hunan Normal University), Changsha, China; ^4^Department of Radiology, The Affiliated Changsha Central Hospital, Hengyang Medical School, University of South China, Changsha, China

**Keywords:** intracerebral hemorrhage, radiomics analysis, hematoma expansion, nomogram, computed tomography

## Abstract

**Background and purpose:**

Hematoma expansion (HE) is a critical event following acute intracerebral hemorrhage (ICH). We aimed to construct a non-contrast computed tomography (NCCT) model combining clinical characteristics, radiological signs, and radiomics features to predict HE in patients with spontaneous ICH and to develop a nomogram to assess the risk of early HE.

**Materials and methods:**

We retrospectively reviewed 388 patients with ICH who underwent initial NCCT within 6 h after onset and follow-up CT within 24 h after initial NCCT, between January 2015 and December 2021. Using the LASSO algorithm or stepwise logistic regression analysis, five models (clinical model, radiological model, clinical-radiological model, radiomics model, and combined model) were developed to predict HE in the training cohort (*n* = 235) and independently verified in the test cohort (*n* = 153). The Akaike information criterion (AIC) and the likelihood ratio test (LRT) were used for comparing the goodness of fit of the five models, and the AUC was used to evaluate their ability in discriminating HE. A nomogram was developed based on the model with the best performance.

**Results:**

The combined model (AIC = 202.599, χ2 = 80.6) was the best fitting model with the lowest AIC and the highest LRT chi-square value compared to the clinical model (AIC = 232.263, χ2 = 46.940), radiological model (AIC = 227.932, χ2 = 51.270), clinical-radiological model (AIC = 212.711, χ2 = 55.490) or radiomics model (AIC = 217.647, χ2 = 57.550). In both cohorts, the nomogram derived from the combined model showed satisfactory discrimination and calibration for predicting HE (AUC = 0.900, sensitivity = 83.87%; AUC = 0.850, sensitivity = 80.10%, respectively).

**Conclusion:**

The NCCT-based model combining clinical characteristics, radiological signs, and radiomics features could efficiently discriminate early HE, and the nomogram derived from the combined model, as a non-invasive tool, exhibited satisfactory performance in stratifying HE risks.

## Introduction

Approximately 10–20% of patients with stroke present with spontaneous intracerebral hemorrhage (ICH), which has poor outcomes. Nearly 40% of patients with ICH die within the first month, and about 80% of survivors require long-term care (1, 2). No effective treatments have emerged to deal with ICH. Hematoma expansion (HE), defined as an increase in hematoma volume after the initial diagnosis of ICH by brain imaging, occurs in approximately one-third of patients with ICH and can predict early neurological deterioration and poor long-term clinical outcomes (3, 4). Recent studies have found that HE can be modified after admission, thus making it a magnet for researchers in this field (3, 5–7). Early and accurate identification of HE in patients with ICH can facilitate individualized treatment.

A variety of clinical and radiological predictors of HE have been reported, such as the time to initial CT, baseline hematoma volume, warfarin use, spot sign, and blend sign. The spot sign derived from CTA has been proven reliable (7), and incorporated into many models to predict HE (8–10). However, CTA examination is expensive, inaccessible in poverty-stricken regions, and not suitable for patients with contraindications, such as contrast reaction or renal impairment. Researchers have also developed some models based on non-contrast CT (NCCT) markers, such as blend sign, swirl sign, island sign, and hypodensity (11, 12). However, these markers show overlapped definitions, and a consensus of diagnostic criteria for HE lacks, all limiting the sensitivity of previous models in predicting the risk of HE (13–15).

Radiomics provides a reproducible, objective, and non-invasive method for the assessment of intralesional heterogeneity, by high-throughput extraction of quantitative features from routine medical images (16–18). Radiomic features from NCCT images can be employed to predict HE (19–21). Based on the scores of radiomic features extracted from NCCT images, the models show good performances in predicting HE (22–26).

However, few studies have evaluated the predictive ability of the model integrating clinical characteristics, radiological signs, and radiomic features of hematoma. In this study, we established a model of this kind and analyzed its performance. Additionally, we generated an individualized nomogram to assess the risk of HE in patients with acute ICH.

## Methods

### Patient selection

**Figure 1:**
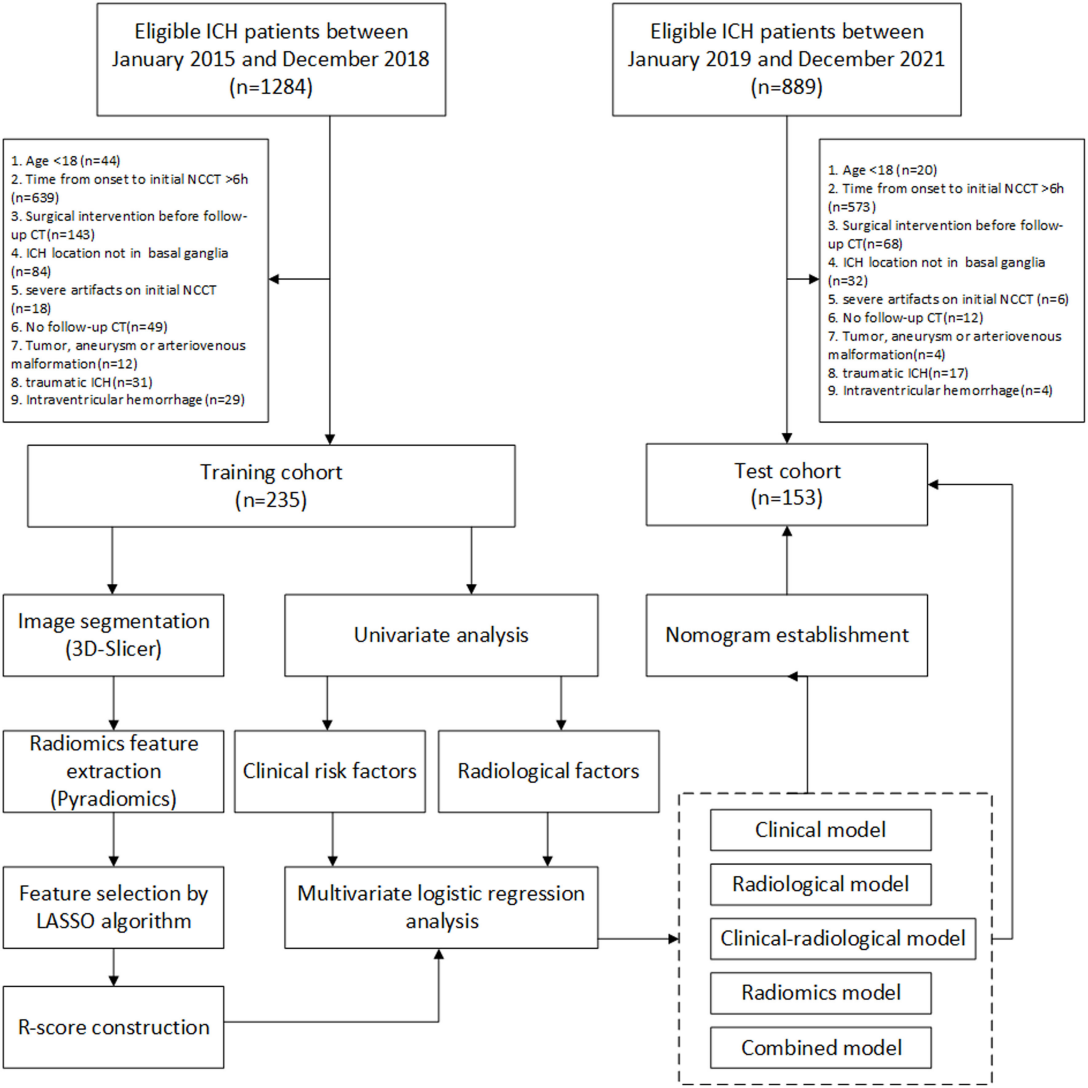
The workflow of this study. LASSO, least absolute shrinkage and selection operator; NCCT, non-contrast computed tomography. ICH, intracerebral hemorrhage.

The workflow is shown in Figure 1. Patients with spontaneous ICH older than 18 years who presented to the Emergency Department of The Affiliated Changsha Central Hospital between January 2015 and December 2021 were retrospectively evaluated. Eligible patients recruited between January 2015 and December 2018 were selected as the training cohort, and patients recruited between January 2019 and December 2021 as the test cohort. The training and test cohorts were then divided based on whether HE was present or not. HE was defined as an absolute increase of 6 ml or a relative increase of 33% in the hematoma volume from initial to follow-up CT, as previously reported (27). The patients were enrolled in our study if the initial cranial CT was performed within 6 h after symptom onset and the follow-up CT was performed within 24 h after the initial CT. Patients who met the following criteria were excluded: (1) surgical intervention prior to follow-up CT, (2) ICH not located in the basal ganglia, (3) severe artifacts on the initial NCCT, (4) no follow-up cranial CT, (5) tumor, aneurysms, or arteriovenous malformation assumed to be the cause of hemorrhage, (6) traumatic ICH, and (7) primary or secondary intraventricular hemorrhage.

### Image data acquisition

The patient was scanned by a 64-slice spiral CT scanner (LightSpeed VCT, GE Healthcare) or a 16-slice spiral CT scanner (Brilliance, Philips Medical System). The scanning energy was set at 120 or 100 kV tube voltage and 350 mA or 400 mA tube current. The slice thickness was 5.0 mm, the pixel spacing was 0.45^*^0.45 mm^2^ or 0.50^*^0.50 mm^2^, and the spacing between slices was 5.0 mm. The image matrix size was 512^*^512. In order to reduce the discrepancy between imaging parameters obtained by different devices, we re-sampled all voxels before extraction into 1.0^*^1.0^*^1.0 mm^3^.

### Lesion segmentation

Cranial NCCT images transferred from the picture archiving and communication system (PACS) were saved as DICOM files, then imported into software (3D Slicer, Version 4.8.1, Harvard University) for semi-automatic segmentation. Two radiologists (Junjie Zhou and Jingjiang Yao) blinded to patients' clinical data manually delineated hematoma regions on NCCT images.

### Radiomic feature extraction

The segmented regions of hematoma were transformed into NRRD format files. Then, a total of 1,409 radiomic features were extracted automatically for each patient using the Pyradiomics software V2.2.0 (28). These features were divided into seven classes (Supplementary Table S1): (1) first-order statistics, (2) shape, (3) gray level co-occurence matrix (GLCM), (4) gray level run length matrix (GLRLM), (5) gray level size zone matrix (GLSZM), (6) neighboring gray tone difference matrix (NGTDM), and (7) gray level dependence matrix (GLDM). All the feature classes, except for shape (a feature class only in original images), were extracted from both the original and derived images, including gradient, exponential, logarithmic, local binary pattern (LBP), square, square root, and wavelet-filtered images. Each feature was named by concatenating the image type from which the feature was extracted, feature class, and feature name with an underline. For example, Wavelet-HHL_GLRLM_ShortRunEmphasis was a feature derived from a wavelet-HHL filtered image, classified into GLRLM, and named as ShortRunEmphasis.

### Radiomics model construction

To construct the radiomics model, we used the least absolute shrinkage and selection operator (LASSO) algorithm to select potential radiomics features in the training cohort. We used 10-fold cross-validation to determine the LASSO tuning parameter (λ) resulting in the maximum area under the receiver operating characteristic curve (AUC). The LASSO forces small coefficients to zero and thereby performs regression and feature selection simultaneously. The radiomics score (R-score) was constructed using features with nonzero coefficients: R-score = (Σβ_j_^*^ X_j_)+Intercept (j = 0,1,2,3……); in the formula, X_j_ represented the j^th^ selected feature; and β_j_ was its coefficient; using the same method, we calculated the corresponding R-score in the test cohort.

### Radiological model construction

Two experienced neuroradiologists (with 12 and 10 years of experience, respectively) who were blinded to the clinical information reviewed radiological features of the initial NCCT images. Discrepancies were resolved by a third investigator. The radiological features included: (1) shape (regular or irregular); (2) density (homogeneous or heterogeneous); (3) swirl sign (absent or present); (4) blend sign (absent or present); (5) black hole sign (absent or present); (6) hypodensity within hematoma (absent or present); (7) island sign (absent or present); (8) satellite sign (absent or present); (9) baseline ICH volume; (10) midline shift; (11) diameter-2D. The radiological features were defined according to previously described radiological criteria (29). In particular, a blend sign was defined as an area of hematoma with low attenuation adjacent to an area of hematoma with high attenuation, with a density differing by at least 18 Hounsfield units between the two areas (30). Hypodensity was defined as a hypodense region within the area of hemorrhage with any shape or dimension, and that was not connected to the surrounding brain parenchyma (31). Midline shift was measured at the level of the septum pellucidum. Diameter-2D was the maximal dimension measured on the largest cross-section of the hematoma. The radiological features were compared between HE and non-HE groups. The radiological model was built by incorporating significant variables into a stepwise logistic regression analysis using the Akaike information criterion (AIC) and likelihood ratio test (LRT) criteria.

### Clinical model construction

Demographic information, medical history, and clinical and laboratory data were collected from the electronic medical record (EMR) system at our hospital. Data of medical history included hypertension, diabetes mellitus, atrial fibrillation, coronary artery disease, renal insufficiency (estimated glomerular filtration rate <60 ml/min/1.73 m^2^), hepatic insufficiency (B and C of Child–Pugh grade), smoking, alcohol consumption, prior ICH, prior ischemic stroke, antiplatelet drugs use, and anticoagulant use. Clinical data included baseline Glasgow Coma Scale (GCS) score, systolic and diastolic blood pressure upon admission, blood pressure targets and duration required to achieve it, time from onset to initial NCCT, and methods of reversal of antiplatelets and anticoagulants. Laboratory data included leukocyte count, neutrophil count, lymphocyte count, neutrophil to lymphocyte ratio (NLR), platelet count, prothrombin time (PT), activated partial thromboplastin time (APTT), international normalized ratio (INR), fibrinogen, baseline blood glucose, serum albumin, aspartate aminotransferase (ALT), alanine aminotransferase (AST), creatinine, blood urea, uric acid, calcium, triglyceride, total cholesterol, low-density lipoprotein cholesterol (LDL-C), and high-density lipoprotein cholesterol (HDL-C). The laboratory testing was performed within 7 h after symptom onset. Demographic information, medical history, and clinical and laboratory data were compared between HE and non-HE groups. The clinical model was constructed by incorporating significant variables into a stepwise logistic regression analysis using the AIC and LRT criteria.

### Clinical-radiological model construction

The clinical-radiological model was formed by incorporating the significant clinical and radiological risk factors into the stepwise logistic regression analysis. The AIC and LRT were also employed as terminal rule during model building.

### Combined model construction

Based on their R-scores, the significant radiological and clinical risk factors were further incorporated into the stepwise logistic regression analysis to form the combined model. The AIC and the LRT were also employed as terminal rule during model building.

### Evaluation of model performance and establishment of nomogram

AIC and LRT were used to compare the goodness of fit of the five models, and receiver operating characteristic (ROC) curve analysis was used to compare their abilities to discriminate HE risk. The AUC of <0.6, 0.6–0.75, and >0.75 indicated poor, acceptable, and satisfactory performances, respectively. The ROC curve analysis was also performed to determine the optimal cut-off value that maximized Youden's index. A number of confusion matrix-related metrics were used to assess the accuracy of the optimal cut-off in each model, including sensitivity, specificity, positive predictive value (PPV), and negative predictive value (NPV). To check the multicollinearity, we calculated the variance inflation factor (VIF) of each trait in each model, where a VIF >5 indicated that multicollinearity was present (32). Based on the most accurate model, a nomogram was developed. Calibration was tested using a calibration plot with bootstraps of 1,000 resamples, which described the degree of fit between actual and nomogram-predicted mortality. Decision curve analysis was used to assess the benefits of the nomogram-assisted decision in a clinical context. Details of the decision curve analysis have been previously reported (33). In addition, we evaluated the performance of the nomogram in predicting in-hospital mortality in the training and test cohorts using ROC curve analysis.

### Statistical analyses

Statistical analysis was performed on R software (version 3.6.3, https://www.r-project.org) and Stata software (version 16.0, STATA Corporation, College Station). Continuous variables were summarized as mean (standard deviation, SD) or median (interquartile range, IQR), and categorical variables as number (percentage). In the univariate analysis, independent *t*-test, Chi-square test, Fisher's exact test, or the Mann–Whitney *U*-test was used. The significant predictors identified in the univariate analysis were tested in the stepwise logistic regression analysis for their relation to HE. AUC values were compared using the DeLong nonparametric method. A two-sided *p* < 0.05 was considered statistically significant. To assess the inter-observer agreement, 50 patients were randomly selected from the entire cohort using stratified sampling and were re-evaluated by the other radiologist using the same method, and then the intraclass correlation coefficient (ICC) was calculated. Good consistency was defined as ICC of 0.75–1, fair consistency as ICC of 0.4–0.75, and poor consistency as ICC < 0.4.

## Results

### Baseline characteristics

In total, 388 patients were analyzed, including 235 in the training cohort and 153 in the test cohort. Table 1 presents the demographic characteristics compared between patients with and without HE. Age and sex did not differ significantly between patients with and without HE in any cohort or between cohorts (all *p* > 0.05). The incidence of HE was 26.4% (62 of 235) in the training cohort and 26.8% (41 of 153) in the test cohort, without a significant difference between the two cohorts (*p* = 0.928). Patients in the training cohort had higher systolic blood pressure (SBP) (165.23 ± 23.55 vs. 160.13 ± 22.4; *p* = 0.035) and higher diastolic blood pressure (DBP) (95.82 ± 17.07 vs. 91.62 ± 15.13; *p* = 0.014) than those in the test cohort.

**Table 1:** Demographic and clinical characteristics of patients with and without HE.

**Variable**	**Training cohort**			**Test cohort**			
	**HE** **(*****n*** = **62)**	**Non-HE** **(*****n*** = **173)**	***P*** **-value**	**HE** **(*****n*** = **41)**	**Non-HE** **(*****n*** = **112)**	***P*** **-value**	***P*** **-value** ^†^
Age, year, mean (SD)	61.50 (13.77)	58.51 (12.09)	0.109	59.68 (12.87)	58.87 (12.44)	0.722	0.871
Male, *n* (%)	47 (75.81)	119(68.79)	0.298	30 (73.17)	67 (59.82)	0.129	0.136
**Medical history**							
Hypertension, *n* (%)	60 (96.77)	163 (94.22)	0.737	37 (90.24)	103 (91.96)	0.748	0.184
Diabetes mellitus, *n* (%)	15 (24.19)	26 (15.03)	0.103	6 (14.63)	21 (18.75)	0.554	0.960
AF, *n* (%)	5 (8.06)	4 (2.31)	0.057	3 (7.32)	3 (2.68)	0.190	0.963
Coronary heart disease, *n* (%)	10 (16.13)	20 (11.56)	0.355	5 (12.20)	12 (10.71)	0.777	0.625
Renal insufficiency, n(%)	3 (4.84)	7 (4.05)	0.726	2 (4.88)	6 (5.36)	1.000	0.792
Hepatic insufficiency, *n* (%)	2(3.23)	4 (2.31)	0.656	2 (4.88)	3 (2.68)	0.611	0.758
Smoking, *n* (%)	21 (33.87)	51 (29.48)	0.520	13 (31.71)	26 (23.21)	0.286	0.273
Alcohol consumption, *n* (%)	11 (17.74)	18 (10.40)	0.132	7 (11.11)	10 (8.93)	0.156	0.714
Prior ICH, *n* (%)	2 (3.23)	6 (3.47)	1.000	3 (7.32)	4 (3.57)	0.386	0.559
Prior ischemic stroke, *n* (%)	2 (3.23)	3 (1.73)	0.609	1 (2.44)	4 (3.57)	1.000	0.524
Antiplatelet drugs use, *n* (%)	8 (12.90)	13 (7.51)	0.202	5 (12.20)	13 (11.61)	1.000	0.365
Anticoagulant use, *n* (%)	4 (6.45)	3 (1.73)	0.081	3 (7.32)	3 (2.68)	0.343	0.461
Baseline GCS score, median [IQR]	12 [9, 13]	13 [12, 14]	<0.001	12 [9, 13]	13 [12, 14]	<0.001	0.229
Baseline GCS score ≤ 8, *n* (%)	12 (19.35)	8 (4.62)	<0.001	9 (21.95)	6(5.36)	0.005	0.664
SBP, mm Hg, mean (SD)	166.56 (23.94)	164.75 (23.46)	0.604	164.73 (21.75)	158.44 (22.69)	0.127	0.035
DBP, mm Hg, mean (SD)	94.26 (19.33)	96.38 (16.21)	0.443	91.98 (15.65)	91.49 (15.01)	0.864	0.014
Blood-pressure-lowering treatment, *n* (%)	20 (32.26)	49 (28.32)	0.559	12 (29.27)	21 (18.75)	0.161	0.088
Time to initial NCCT, hour, median [IQR]	1.44 [1.08, 2.56]	2.54 [1.65, 4.49]	<0.001	1.49 [0.97, 2.14]	2.51 [1.66, 4.48]	<0.001	0.679
Time to initial NCCT ≤ 3 h, *n* (%)	51(82.26)	97(56.07)	<0.001	36 (87.80)	63 (56.25)	<0.001	0.730
Death within the hospital	8(12.90)	1(0.58)	< 0.001	5(12.20)	0(0.00)	0.001	0.772

† Comparison between the training cohort and test cohort. AF, Atrial fibrillation; DBP, diastolic blood pressure; GCS, Glasgow Coma Scale; HE, hematoma expansion; IQR, interquartile range; NCCT, non-contrast computed tomography; SBP, systolic blood pressure.

### Clinical model

Tables 1, 2 illustrate the clinical data and laboratory tests compared between patients with and without HE. In the training cohort, the following variables were significantly associated with HE: baseline GCS (*p* < 0.001), time to initial NCCT (*p* < 0.001), leukocyte count (*p* < 0.001), neutrophil count (*p* < 0.001), lymphocyte count (*p* = 0.020), NLR (*p* < 0.001), platelet count (*p* = 0.049), total cholesterol (*p* = 0.017), and LDL-C (*p* = 0.006). In the multivariate analysis, three clinical factors independently associated with HE were used to construct the clinical model, consisting of baseline GCS score (OR = 0.80; 95% CI = 0.70–0.91; *p* = 0.001), NLR (OR = 1.07; 95% CI = 1.03–1.12, *p* = 0.001), and time to initial NCCT (≤3 vs. >3 h; OR = 3.28; 95% CI = 1.50–7.19, *p* = 0.003).

**Table 2:** Comparisons of laboratory tests between patients with and without HE.

**Variable**	**Training cohort**			**Test cohort**			
	**HE**	**Non-HE**	***P*** **-value**	**HE**	**Non-HE**	***P*** **-value**	***P*** **-value** ^†^
	**(*****n*** = **62)**	**(*****n*** = **173)**		**(*****n*** = **41)**	**(*****n*** = **112)**		
Leukocyte, *10^∧^9/L, median [IQR]	9.80 [8.22, 13.39]	8.30 [6.19, 10.23]	< 0.001	9.84 [7.80, 12.94]	7.91 [6.26, 9.52]	< 0.001	0.376
Neutrophil count, *10^∧^9/L, median [IQR]	8.09 [6.48, 10.84]	6.34 [4.20, 8.38]	< 0.001	8.15 [6.23, 10.79]	5.81 [4.27, 7.76]	< 0.001	0.366
Lymphocyte count, *10^∧^9/L, median [IQR]	1.04 [0.55, 1.65]	1.27 [0.93, 1.57]	0.020	0.84 [0.54, 1.35]	1.35 [0.95, 1.69]	< 0.001	0.656
NLR, median [IQR]	10.10 [6.64, 16.21]	4.72 [3.11, 7.68]	< 0.001	9.91 [6.33, 18.27]	4.26 [2.64, 7.42]	< 0.001	0.385
Platelet count, *10^∧^9/L, median [IQR]	182.50 [146.00, 231.00]	197.00 [169.00, 238.00]	0.049	206.00 [154.00, 232.00]	200.50 [167.50, 233.00]	0.709	0.511
PT, s, median [IQR]	10.70 [10.30, 11.60]	10.80 [10.30, 11.40]	0.769	10.90 [10.60, 12.20]	10.70 [10.30, 11.30]	0.084	0.947
APTT, s, median [IQR]	26.20 [23.40, 29.60]	26.00 [23.80, 28.40]	0.553	26.80 [24.20, 29.80]	26.14 [23.95, 28.35]	0.249	0.653
INR, median [IQR]	1.00 [0.92, 1.04]	1.00 [0.92, 1.03]	0.850	1.00 [0.96, 1.07]	1.00 [0.93, 1.01]	0.083	0.885
Fibrinogen, g/L, median [IQR]	2.66 [2.24, 3.50]	2.70 [2.24, 3.20]	0.931	2.60 [2.18, 3.10]	2.70 [2.34, 3.30]	0.231	0.741
Baseline glucose level, mmol/L, median [IQR]	6.55 [5.90, 8.20]	6.70 [5.70, 7.60]	0.531	6.00 [5.20, 7.80]	6.85 [5.90, 7.85]	0.055	0.914
Serum albumin level, g/L, mean (SD)	42.76 (4.22)	43.26 (4.51)	0.421	42.73 (4.75)	43.52 (3.58)	0.334	0.676
ALT, U/L, median [IQR]	20.50 [16.00, 33.00]	19.00 [14.00, 30.00]	0.165	21.00 [15.00, 35.00]	20.00 [15.00, 29.50]	0.584	0.625
AST, U/L, median [IQR]	25.00 [20.00, 32.00]	25.00 [21.00, 29.90]	0.913	25.00 [21.00, 30.91]	25.00 [21.50, 31.00]	0.490	0.621
Creatinine, μmol/L, median [IQR],	72.50 [61.00, 92.00]	73.00 [59.00, 87.00]	0.795	72.00 [60.00, 95.00]	67.00 [55.00, 80.00]	0.143	0.067
Blood urea nitrogen, mmol/L, median [IQR]	5.32 [4.33, 6.89]	5.62 [4.52, 6.58]	0.509	5.48 [4.87, 6.12]	5.56 [4.50, 6.44]	0.856	0.750
Blood uric acid, μmol/L, median [IQR]	348.50 [276.00, 414.00]	330.00 [260.00, 399.00]	0.108	310.00 [264.00, 386.00]	330.00 [257.00, 387.50]	0.926	0.244
Calcium, mmol/L, mean(SD)	2.31 (0.13)	2.33 (0.12)	0.3312	2.30 (0.13)	2.34 (0.12)	0.089	0.838
Total cholesterol, mmol/L, mean (SD)	4.31 (0.98)	4.64 (0.91)	0.017	4.31 (0.87)	4.61 (0.95)	0.079	0.838
Triglyceride, mmol/L, median [IQR]	1.32 [0.97, 2.59]	1.49 [1.00, 2.42]	0.876	1.16 [0.86, 1.81]	1.39 [1.00, 1.96]	0.145	0.218
LDL-C, mmol/L, mean (SD)	2.39 (0.75)	2.71 (0.78)	0.006	2.37 (0.71)	2.65 (0.79)	0.048	0.538
HDL-C, mmol/L, mean (SD)	1.15 (0.36)	1.23 (0.36)	0.135	1.24 (0.43)	1.27 (0.34)	0.654	0.182

† Comparison between the training cohort and test cohort. HE, hematoma expansion. IQR, interquartile range; PT, prothrombin time; APTT, activated partial thromboplastin time; INR, international normalized ratio; ALT, aspartate aminotransferase; AST, alanine aminotransferase; LDL-C, low-density lipoprotein cholesterol; HDL-C, high-density lipoprotein cholesterol.

### Radiological model

The univariate analysis of the training cohort revealed significant predictors of HE (Table 3): diameter-2D (*p* < 0.001), midline shift (*p* < 0.001), baseline ICH volume (*p* < 0.001), irregular shape (*p* = 0.004), heterogeneous density (*p* < 0.001), swirl sign (*p* < 0.001), blend sign (*p* = 0.003), black hole sign (*p* = 0.007), hypodensity (*p* < 0.001), island sign (*p* = 0.030), and satellite sign (*p* = 0.002). Through the stepwise logistic regression analysis, three radiological risk factors independently associated with HE were filtered out to construct the radiological model, consisting of blend sign (OR = 3.85; 95% CI = 1.73–8.53, *p* = 0.001), hypodensity (OR = 4.59; 95% CI = 2.35–8.95; *p* < 0.001), and midline shift (OR = 1.26; 95% CI = 1.08–1.46; *p* = 0.003) (Table 4).

**Table 3:** Comparison of radiological characteristics and R-score between patients with and without HE.

**Variable**	**Training cohort**			**Test cohort**			
	**HE**	**Non-HE**	***P*** **-value**	**HE**	**Non-HE**	***P*** **-value**	***P*** **-value** ^†^
	**(*****n*** = **62)**	**(*****n*** = **173)**		**(*****n*** = **41)**	**(*****n*** = **112)**		
Diameter-2D, cm, median [IQR]	4.62 [3.31, 5.74]	3.59 [2.83, 4.60]	< 0.001	4.30 [2.73, 5.51]	3.37 [2.72, 4.46]	0.023	0.137
Middle shift, mm, median [IQR]	0 [0, 3.89]	0 [0, 0)	< 0.001	0 [0, 3.40]	0 [0, 0]	0.003	0.996
Baseline ICH volume, ml, median [IQR]	17.60 [9.51, 26.92]	8.30 [4.79, 15.88]	< 0.001	16.18 [7.36, 26.92]	7.24 [3.74, 14.54]	0.001	0.099
Baseline ICH volume, *n* (%)			<0.001			0.001	0.561
≤15	26 (41.94)	123 (71.10)		19 (46.34)	86 (76.79)		
16–29	24 (38.71)	41 (23.70)		15 (36.59)	22 (19.64)		
≥30	12 (19.35)	9 (5.20)		7 (17.07)	4 (3.57)		
Irregular shape, *n* (%)	33 (53.23)	56 (32.37)	0.004	18 (43.90)	32 (28.57)	0.073	0.297
Heterogeneous, *n* (%)	39 (62.90)	47 (27.17)	< 0.001	23 (56.10)	32 (28.57)	0.002	0.897
Swirl sign, *n* (%)	36 (58.06)	44 (25.43)	< 0.001	22 (53.66)	25 (22.32)	< 0.001	0.495
Blend sign, *n* (%)	16 (25.81)	18 (10.40)	0.003	10 (24.39)	8 (7.14)	0.008	0.445
Black hole sign, *n* (%)	19 (30.65)	26 (15.03)	0.007	12 (29.27)	10 (8.93)	0.001	0.224
Hypodensity, *n* (%)	41 (66.13)	50 (28.90)	< 0.001	23 (56.10)	28 (25.00)	< 0.001	0.281
Island sign, *n* (%)	9 (14.52)	10 (5.78)	0.03	6 (14.63)	6 (5.36)	0.086	0.932
Satellite sign, *n* (%)	30 (48.39)	47 (27.17)	0.002	16 (39.02)	31 (27.68)	0.178	0.673
R-score, median (IQR)	−0.68 [−1.12, −0.32]	−1.28 [−1.58, −0.95]	< 0.001	−0.84 [−1.29, −0.26]	−1.34 [−1.62, −1.12]	< 0.001	0.1651

† Comparison between the training cohort and test cohort. HE, hematoma expansion. IQR, interquartile range. Diameter-2D was the maximal dimension measured on the largest cross-section of the hematoma. Midline shift was measured at the level of the septum pellucidum. ICH volume was calculated by the formula A^*^ B^*^ C/2.

**Table 4:** Construction and comparison of five HE prediction models in the training cohort.

	**Adjusted OR (95% CI)**	***P*** **Value**	**AIC**	**LRT (χ^2^)**	**VIF**
**Clinical model**			232.263	46.940	
GCS score	0.80 (0.70–0.91)	0.001			1.15
NLR	1.07 (1.03–1.12)	0.001			1.11
Time to baseline NCCT (≤3 vs. >3 h)	3.28 (1.50–7.19)	0.003			1.04
**Radiological model**			227.932	51.270	
Blend sign	3.85 (1.73–8.53)	0.001			1.05
Hypodensity	4.59 (2.35–8.95)	< 0.001			1.03
Middle shift	1.26 (1.08–1.46)	0.003			1.08
**Clinical-radiological model**			212.711	55.490	
Time to baseline NCCT (≤3 vs. >3 h)	3.79 (1.68–8.53)	0.001			1.01
NLR	1.08 (1.04–1.13)	< 0.001			1.05
Hypodensity	4.56 (2.27–9.17)	< 0.001			1.04
Blend sign	4.86 (2.09–11.29)	< 0.001			1.02
**Radiomics model**			217.647	57.550	
R-score	7.62 (3.94–14.73)	< 0.001			
**Combined model**			202.599	80.600	
Time to baseline NCCT (≤3 vs. >3 h)	3.56 (1.56–8.13)	0.003			1.02
NLR	1.06 (1.01–1.11)	0.011			1.18
Hypodensity	2.93 (1.39–6.20)	0.005			1.22
Blend sign	3.64 (1.56–8.50)	0.003			1.07
R-score	3.72 (1.72–8.06)	0.001			1.47

AIC, Akaike information criterion; CI, confidence interval; GCS, Glasgow Coma Scale; LRT, likelihood ratio test; NLR, neutrophil to lymphocyte ratio; OR odds ratio; NCCT, non-contrast computed tomography, and VIF, variance inflation factor. Predictive model with a lower AIC value and a higher LRT χ^2^ values represented the better model with better goodness of fit and relative strength.

### Clinical-radiological model

Based on the stepwise logistic regression analysis of clinical and radiological risk factors, four independent risk factors associated with HE were used to construct the clinical-radiological model (Table 4), including time to initial NCCT (≤3 vs. >3 h; OR = 3.79; 95% CI = 1.68–8.53; *p* = 0.001), NLR (OR = 1.08; 95% CI = 1.04–1.13, *p* < 0.001), hypodensity (OR = 4.56; 95% CI = 2.27–9.17, *p* < 0.001), and blend sign (OR = 4.86; 95% CI = 2.09–11.29, *p* < 0.001).

### R-score and radiomics model

**Figure 2:**
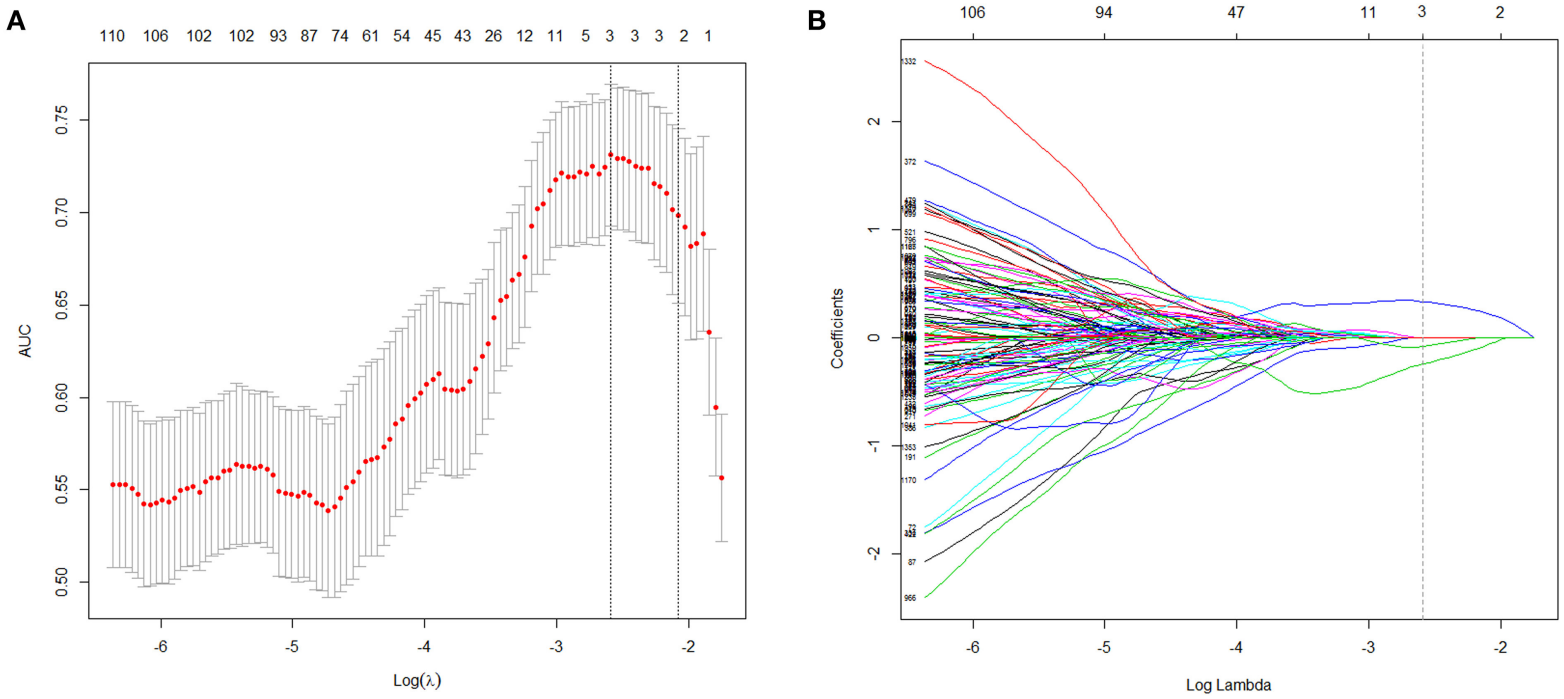
Radiomics features selection with LASSO. This method minimized the sum of residues, with the sum of the absolute values of the selected features coefficients being not more than a tuning parameter (λ). **(A)** Tuning parameter (λ) selection in the LASSO model used 10-fold cross-validation *via* maximum criteria. The area under the curves (AUC) was plotted vs. log(λ). The dotted vertical lines were drawn at the optimal values using maximum and 1-SE criteria. A log(λ) value of −2.59 was opted (maximum criteria). **(B)** LASSO coefficient profiles (y-axis) of the features. Each colored line represents the coefficient of each feature. The upper and lower x-axis represented the features number and the log(λ), respectively. The dashed vertical line was drawn at the value chosen using 10-fold cross-validation in the log(λ) sequence, and three features with nonzero coefficients were indicated. LASSO, least absolute shrinkage and selection operator; SE, standard error.

**Figure 3:**
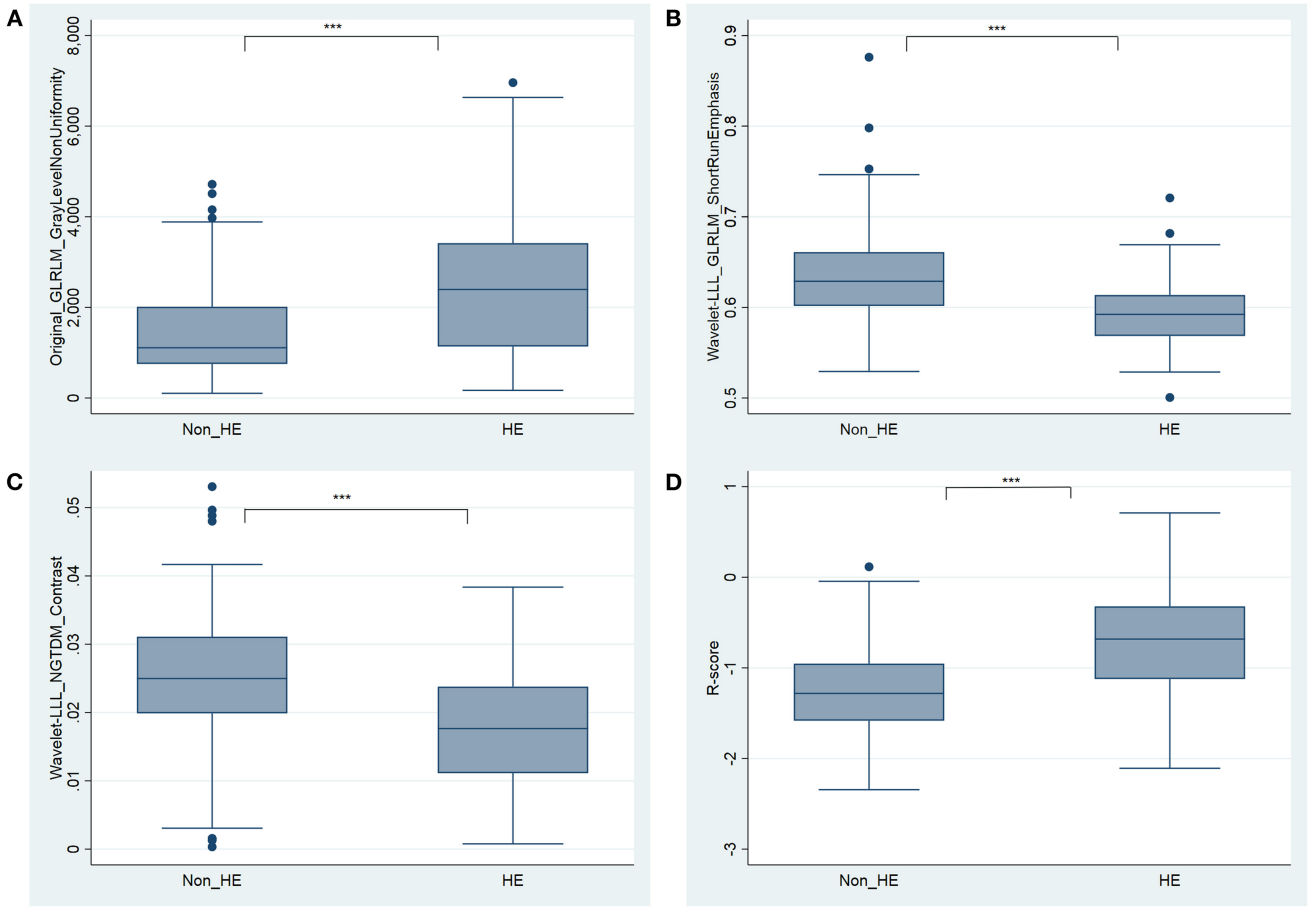
Boxplots for the selected features [**(A)** Original_GLRLM_GrayLevelNonUniformity, **(B)** Wavelet-LLL_GLRLM_ShortRunEmphasis, and **(C)** Wavelet-LLL_NGTDM_Contrast] and the constructed R-score **(D)** between HE and non-HE groups. *** significant at *p* < 0.001. HE, hematoma expansion.

The R-score was derived by selecting three features after dimension reduction using the LASSO algorithm (Figure 2). The calculation formula was as follows: R-score = 1.8476 + 0.0003^*^ Original_GLRLM_GrayLevelNonUniformity-5.178^*^ Wavelet-LLL_GLRLM_ShortRunEmphasis– 7.895^*^Wavelet-LLL_NGTDM_Contrast. Figure 3 shows the distributions of the selected features in patients with HE and non-HE (all *p* < 0.001). Patients with HE had a higher R-score than patients with non-HE (*p* < 0.001). R-score (OR = 7.62; 95% CI = 3.94–14.73; *p* < 0.001) was significantly related to HE (Table 4).

### Combined model

Based on the stepwise logistic regression analysis of clinical and radiological risk factors and R-score, five independent risk factors associated with HE were used to construct the combined model (Table 4), including time to initial NCCT (≤3 vs. >3 h; OR = 3.56; 95% CI = 1.56–8.13; *p* = 0.003), NLR (OR = 1.06; 95% CI = 1.01–1.11, *p* = 0.011), hypodensity (OR = 2.93; 95% CI = 1.39–6.20, *p* = 0.005), blend sign (OR = 3.64; 95% CI = 1.56–8.50, *p* = 0.003), and R-score (OR = 3.72; 95% CI = 1.72–8.06; *p* = 0.001). The mean variance inflation factor for the five predictors was 1.19 (range 1.02–1.47), which suggested no multicollinearity.

### Between-model comparison and nomogram establishment

**Figure 4:**
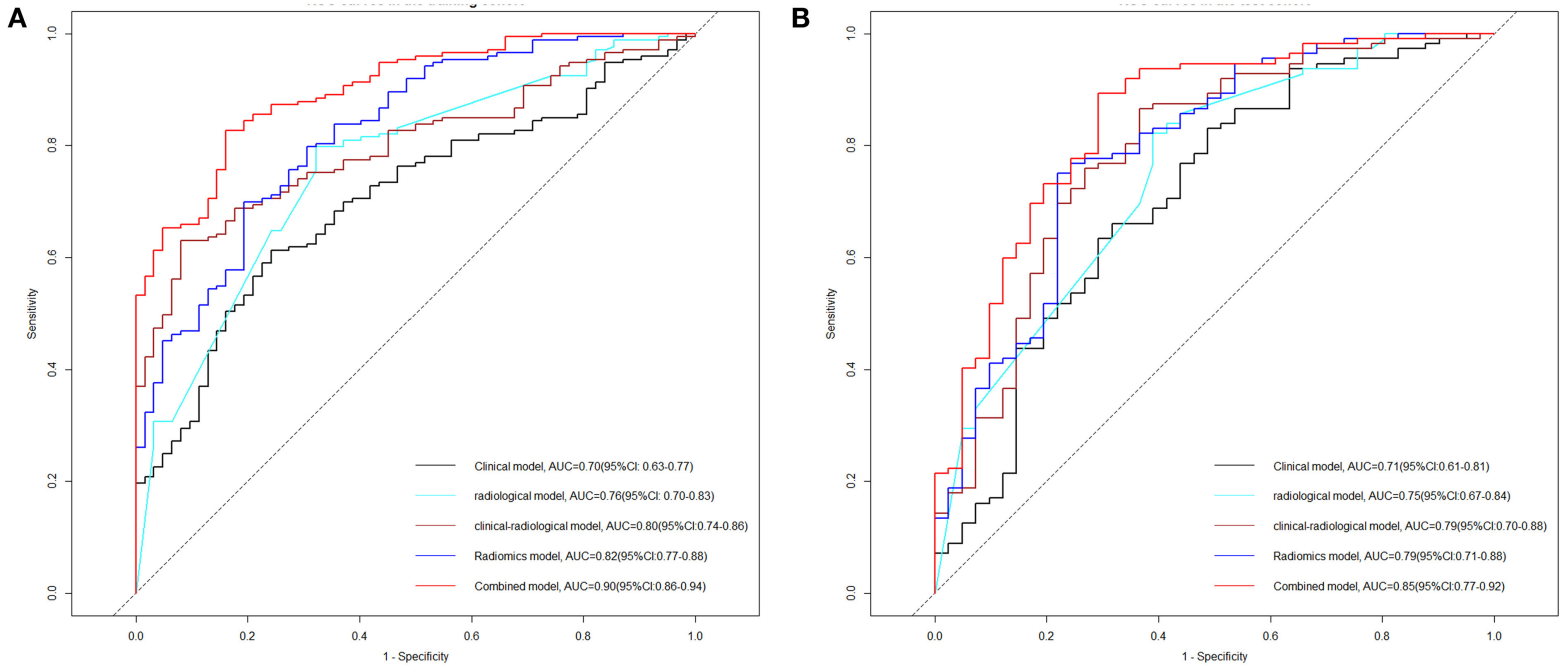
Receiver operating characteristic (ROC) curve analyses of the five models on HE in the training cohort **(A)** and test cohort **(B)**. HE, hematoma expansion; AUC, area under the curve; CI, confidence interval.

As shown in Table 4, the combined model (AIC = 202.599, χ^2^ = 80.6) achieved the lowest AIC and the highest LRT chi-square value, compared to the clinical model (AIC = 232.263, χ^2^ = 46.940), radiological model (AIC = 227.932, χ^2^ = 51.270), clinical-radiological model (AIC = 212.711, χ^2^ = 55.490), and radiomics model (AIC = 217.647, χ^2^ = 57.550). Therefore, this combined model was determined to be the best fitting model. The performances of five models in training and test cohorts are shown in Table 5 and Figure 4. The combined model demonstrated a satisfactory ability in discriminating HE, with an AUC of 0.90 (95%CI: 0.86–0.94) in the training cohort, and an AUC of 0.85 (95%CI: 0.77–0.92) in the test cohort. Conversely, in both cohorts, the clinical, radiological, and clinical-radiological models showed lower discrimination ability for HE as compared with the combined model (*P* < 0.05). With the optimal cut-off, the combined model's sensitivity, specificity, PPV, and NPV were 83.87%, 82.66%, 63.42%, and 93.46% in the training cohort, and 80.10%, 89.29%, 73.25%, and 92.46% in the test cohort, respectively.

**Table 5:** Performance comparison of five models in the training and test cohorts.

	**Cut-off^†^**	**AUC(95%CI)**	**Sensitivity**	**Specificity**	**Accuracy**	**PPV**	**NPV**
**Training cohort**							
Clinical model	0.21	0.70 (0.63–0.77) ^**^	75.81%	61.27%	65.11%	41.23%	87.60%
Radiological model	0.29	0.76 (0.70–0.83) ^**^	67.74%	79.77%	76.60%	54.55%	87.34%
Clinical-radiological model	0.18	0.80 (0.74–0.86)^**^	78.94%	80.01%	79.73%	58.60%	91.38%
Radiomics model	0.22	0.82 (0.77–0.88) ^**^	80.65%	69.94%	72.77%	49.02%	90.98%
Combined model	0.30	0.90 (0.86–0.94)	83.87%	82.66%	82.98%	63.42%	93.46%
**Test cohort**							
Clinical model	0.20	0.71(0.61–0.81) ^*^	68.29%	66.07%	66.67%	42.42%	85.06%
Radiological model	0.17	0.75 (0.67–0.84) ^**^	70.73%	67.86%	68.63%	44.62%	86.36%
Clinical-radiological model	0.37	0.79 (0.70–0.88)^*^	73.41%	76.61%	75.75%	53.47%	88.73%
Radiomics model	0.22	0.79 (0.71–0.88)	78.05%	75.00%	75.82%	53.33%	90.32%
Combined model	0.31	0.85 (0.77–0.92)	80.10%	89.29%	86.83%	73.25%	92.46%

AUC, area under the curve; CI, confidence interval; NPV, negative predictive value; PPV, positive predictive value.

* p < 0.05 and

** p < 0.01 vs. combined model.

† Determined by maximizing the Youden's Index (Sensitivity+Specificity-1).

**Figure 5:**
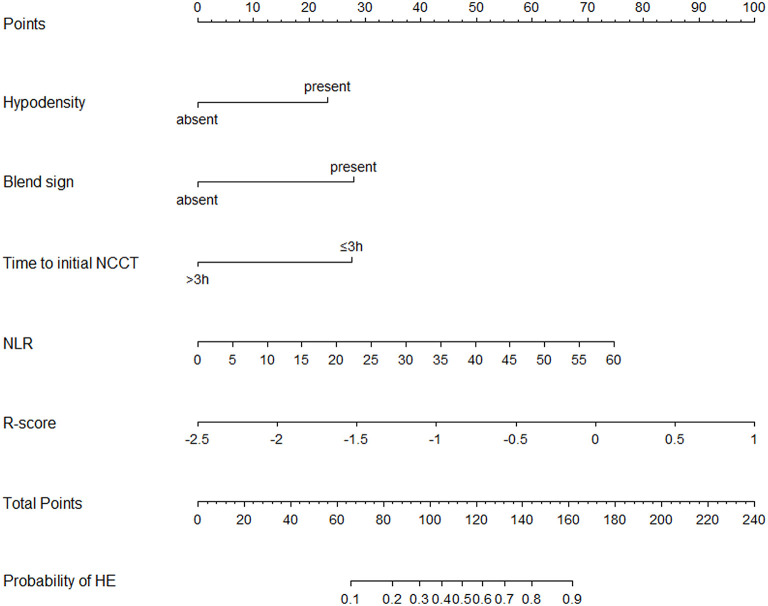
Nomogram for predicting the probability of HE was developed based on the combined model. Points were assigned for hypodensity, blend sign, time to initial NCCT, NLR, and R-score by drawing a line upward from the corresponding values to the “points line.” The “total points” are calculated as the sum of the individual score of each of the five variables included in the nomogram. The risk of HE was determined by drawing a vertical line from the total point axis to the lowest line of the nomogram. NCCT, non-contrast computed tomography; NLR, Neutrophil to lymphocyte ratio; HE, hematoma expansion.

**Figure 6:**
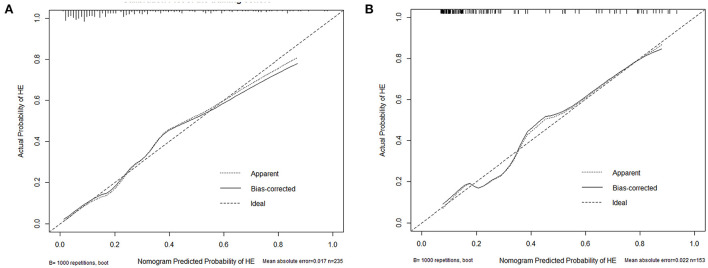
Calibration plot of the nomogram in the training **(A)** and test cohort **(B)**. The dotted line represents the performance of the nomogram, whereas the solid line corrects for any bias in the nomogram. The dashed line represents the reference line where an ideal nomogram would lie.

**Figure 7:**
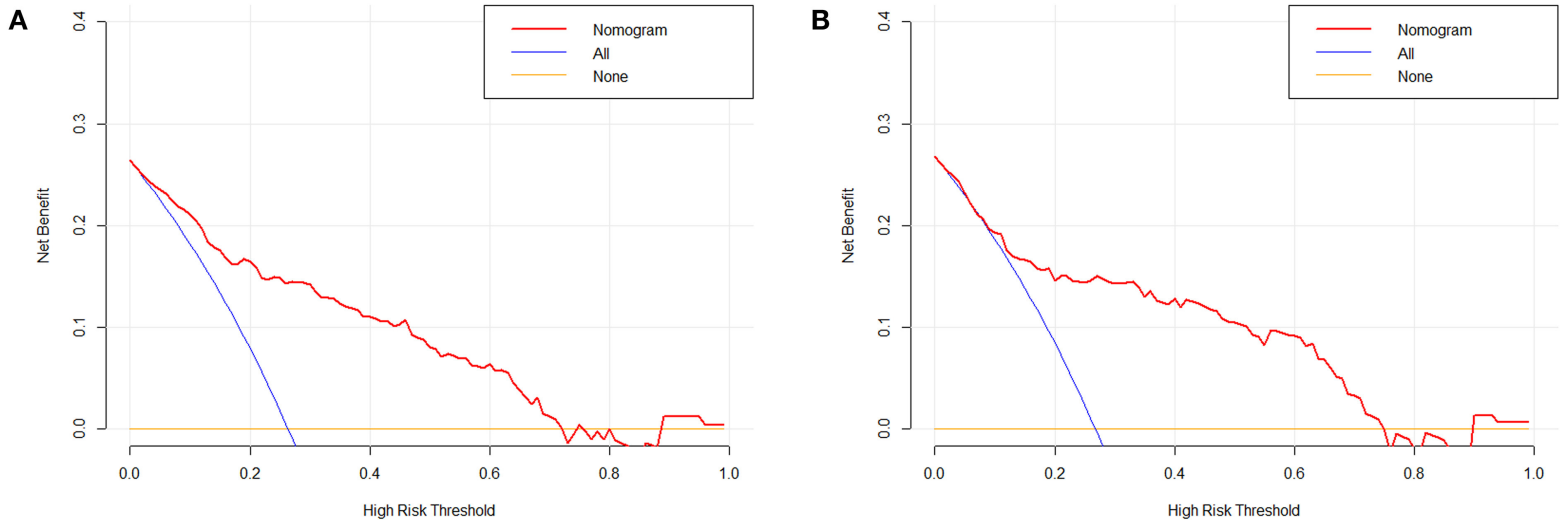
Decision curve analysis of the nomogram in the training **(A)** and test cohort **(B)**. The x-axis indicates the threshold probability. The y-axis measures the net benefit. The blue line displays the net benefit of the strategy of treating all patients. The orange line illustrates the net benefit of the strategy of treating no patients. The red line indicates the nomogram. Decision curve analysis is a specific method developed for evaluating the prognostic value of nomogram strategies.

**Figure 8:**
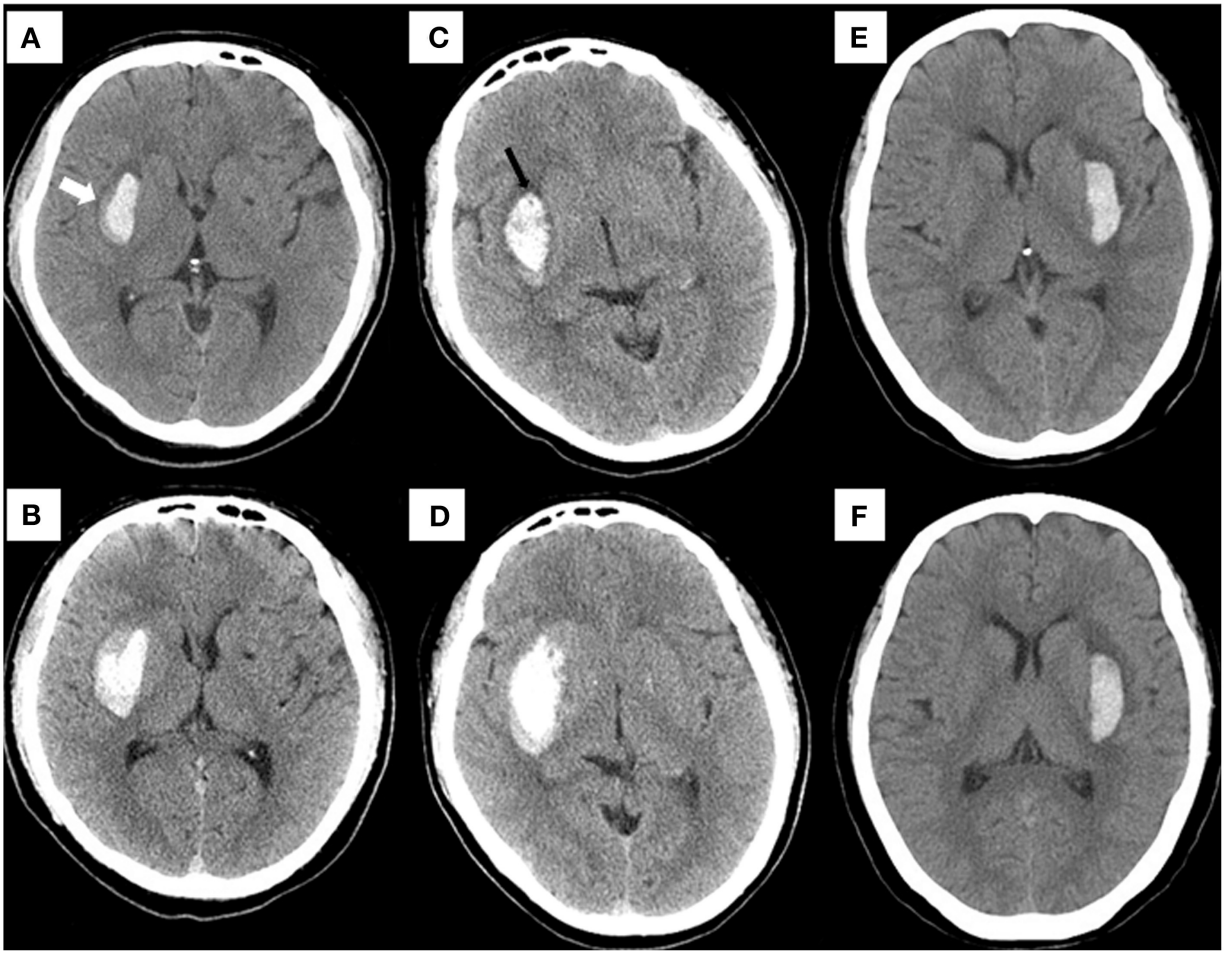
Examples of using the nomogram to predict the risk of HE. Case 1: **(A)** a patient who underwent cranial NCCT within 1.5 h of onset presented with a hematoma (6.1 ml) in the basal ganglia. The risk of HE estimated by nomogram was approximately 71.36% (blend sign = present, hypodensities = absent, NLR = 10.22, time to initial NCCT <3 h, R-score = −0.21, total points = 134, and estimated HE risk = 71.36%). A blend sign (white arrow) can be seen. **(B)** The hematoma volume was enlarged to about 11.8 ml on the follow-up CT at 8 hours. Case 2: **(C)** a patient who underwent cranial NCCT within 2.5 h of onset presented with a hematoma (10.1 ml) in the basal ganglia. The risk of HE estimated by nomogram was approximately 70.78% (blend sign = absent, hypodensities = present, NLR = 13.03, time to initial NCCT <3 h, R-score = −0.19, total points = 131, and estimated HE risk = 70.78%). A hypodensity sign (black arrow) can be seen. **(D)** The hematoma volume was enlarged to about 24.8 ml on the follow-up CT at 10 Hours. Case 3: **(E)** using this nomogram, the hematoma (4.8 ml) on the initial NCCT showed a low expansion risk (blend sign = absent, hypodensities = absent, NLR = 2.52, time to initial NCCT>3 h, R-score = −1.59, total points = 30, and estimated HE risk = 1.99%). **(F)** At 22.5 h after onset, the follow-up CT detected a hematoma of similar size (5.2 ml). NCCT, noncontrast computed tomography. HE, hematoma expansion.

Based on the combined model, a nomogram (Figure 5) was constructed to visualize the risk of HE. The calibration curves demonstrated favorable agreement between the results predicted by the nomogram and observed in the real setting in either the training or the test cohort (Figure 6). In the decision curve analysis, the net benefit of a model can be evaluated by comparing the true-and false-positive results. Here, we performed a decision curve analysis to assess whether the nomogram-assisted decision can improve patient outcomes. As shown in Figure 7, when the threshold probability was 2.0–72.0% in the training cohort, and 2.0–74.0% in the test cohort, the nomogram provided greater net benefits than the “treat all” or “treat none” strategies, which indicates the clinical usefulness of the nomogram. For example, if the threshold probability of a patient was 40% (the patient would opt for treatment if the probability of HE was > 40%), then the net benefit would be 0.111 in the training cohort and 0.129 in the test cohort. Figure 8 demonstrated three cases of using this nomogram for the risk evaluation of HE.

### The performance of the nomogram for predicting in-hospital mortality

**Figure 9:**
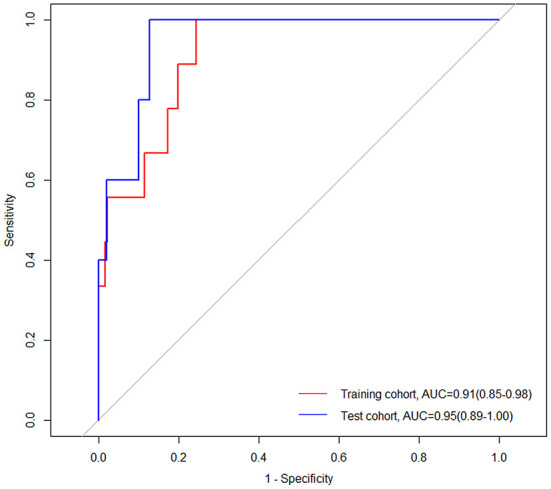
Receiver operating characteristic (ROC) curves of the nomogram for predicting in-hospital mortality in the training and test cohorts. AUC, area under the curve; CI, confidence interval.

Nine patients in the training cohort (3.83%) and five patients in the test cohort (3.27%) died in the hospital (Table 1). The nomogram demonstrated a satisfactory ability in predicting in-hospital mortality, with an AUC of 0.91 (95%CI: 0.85–0.98) in the training cohort, and an AUC of 0.95 (95%CI: 0.89–1.00) in the test cohort (Figure 9).

### Reproducibility

The reproducibility analysis revealed that 1,221 of 1,409 (86.7%) radiomic features had good consistency (ICC ≥ 0.75). The numbers of features with fair consistency (0.75 > ICC ≥ 0.4) and with poor consistency (ICC < 0.4) were 140 (9.9%) and 48 (3.4%), respectively. For the three selected features, the ICC values were 0.95 (95% CI: 0.91–0.97), 0.97 (95% CI: 0.93–0.98), and 0.93 (95% CI: 0.88–0.96), respectively (Supplementary Table S2). The ICC value was 0.85 (95% CI: 0.75–0.91) for blend sign and 0.88 (95% CI: 0.80–0.93) for hypodensity, indicating satisfactory consistency.

## Discussion

In this retrospective study, we built five models (clinical model, radiological model, clinical-radiological model, radiomics model, and combined model) and compared their performances in predicting HE risk. The NCCT model combining clinical characteristics, radiological signs, and radiomics features showed the best performance. The nomogram derived from the combined model was able to predict the risk of HE with good discrimination and calibration. This tool may provide a more individualized strategy for discriminating early HE, particularly for patients with contraindications, such as contrast reaction or renal impairment, or in institutions where CTA is not available.

In our study, time to initial NCCT, NLR, hypodensity, and blend sign were independent risk factors of HE, which is in accordance with the findings previously reported. Several studies have demonstrated that the time from onset to initial NCCT is a strong predictor of HE (8, 9, 11). As a dynamic process, HE represents an intermediate state between initial and final (stabilized) hematoma (34). If admitted earlier after ICH, the patients are more likely to exhibit an unstable hematoma on initial CT, thus leaving a greater chance of detecting HE by follow-up imaging. In addition, our study revealed that NLR, which can be easily and cost-effectively detected, was an independent risk factor of HE. Increasing evidence suggests that inflammatory responses and damage to microvascular integrity are implicated in the pathophysiology of brain injury following ICH (35). Reflecting the balance between neutrophils and lymphocytes, NLR is regarded as an efficient biomarker for systemic inflammation. Alimohammadi et al. (36) have found that NLR is a key predictor of HE in patients with ICH, which is in accordance with our findings.

Several NCCT markers, rather than spot sign which is dependent on CTA, have demonstrated abilities to predict HE. These markers may reflect a similar pathophysiological process, which is manifested as active hemorrhage secondary to vessel rupture at different time points (37). Blend sign (30, 38) and hypodensity (31) have been identified as independent predictors of HE, which is consistent with our results. In previous studies, however, a single sign displayed limitations in predicting HE in clinical settings, as shown by that the blend sign had a low sensitivity, and hypodensity had a low specificity (27, 39).

In this study, three radiomics features were selected from 1,409 candidate features to construct the radiomics model strictly, one of which was extracted from the original image and the other two from wavelet-filtered images. Details of the three selected features are shown in Supplementary Table S2. In accordance with previous literature, these features were associated with variations in morphology and intensity of hematoma (28). These variations may indicate active or multifocal bleeding, both of which are thought to be the primary mechanism underlying HE.

Several prediction systems, independent of CTA spot sign, have been published for predicting HE (11, 12, 22, 24, 37, 40–44) (Supplementary Table S3). However, none has actually improved clinical or research decision-making. Possible factors may explain this situation. First, these systems vary in the definition of HE, the time from onset to initial NCCT, the time of follow-up CT, the location of ICH, thereby making each only suitable for a certain patient population. Second, only a few systems have been validated in external prospective trials, and their accuracy needs to be further investigated. Third, most of these systems were based on the data retrospectively collected from small-sized cohorts in a single center, which increases the risk of selection bias. Fourth, the clinical parameters of some systems were not reported, such as sensitivity and specificity. The nomogram established in our study, which integrated the clinical characteristics, radiology signs, and radiomics features, showed satisfactory performance in discriminating HE (AUC = 0.900, sensitivity = 83.87% in training cohort; AUC = 0.850, sensitivity = 80.10% in test cohort, respectively). Additionally, we enrolled patients with acute ICH located in the deep basal ganglia region, a site that is mostly frequented by hemorrhage, which reduced the bias arising from etiology. Several studies have demonstrated that patients with deep ICH are more susceptible to HE than those with lobar ICH (45–47). Deep ICH is caused primarily by hypertensive vasculopathy, while lobar ICH is more relevant to cerebral amyloid angiopathy (48). Derived from the data of only patients with deep basal ganglia ICH, our nomogram is more applicable and targeted.

This study has some limitations. First, this is a retrospective study with its natural drawbacks. Second, the established nomogram lacks external validation in broader populations outside China. Third, as a single-center study with relatively small sample size, a possible selection bias is not possible to be ruled out. Fourth, several patients with ICH were excluded from the study due to surgical intervention prior to follow-up CT scans, which decreases the proportion of HE. Finally, a relatively large proportion of patients were excluded due to a lack of repeat CT heads, possibly because they remained well or had deteriorated significantly enough to warrant a “do not resuscitate” status. This could represent a relatively stable or significant expansion of the underlying ICH. Despite these limitations, our study has a few strengths, such as blind imaging assessment, systematic assessment of clinical and laboratory characteristics, and easily and quickly obtained predictive factors included in the nomogram.

## Conclusion

An NCCT-derived model combining radiomics, clinical, and radiological features show a satisfactory performance in predicting HE. The nomogram derived from the combined model can individualize the risk of HE, and its high sensitivity and calibration may help clinicians to screen out patients with ICH appropriate for anti-expansion therapy.

## Data availability statement

The raw data supporting the conclusions of this article will be made available by the authors, without undue reservation.

## Ethics statement

The studies involving human participants were reviewed and approved by the Ethical Committee of Affiliated Changsha Central Hospital. The Ethics Committee waived the requirement of written informed consent for participation.

## Author contributions

WX, HG, HL, ZW, and XL designed the study. WX, KS, and FL collected the data. JZ, JY, and ZW reviewed CT images. WX, HG, HL, and KS analyzed and interpreted the data. WX, HG, HL, QD, KS, FL, JZ, JY, ZW, and XL drafted and modified the manuscript. All authors contributed to the article and approved the submitted version.

## Funding

The project was partly supported by the Science and Technology Research Plan of Hunan Provincial Health Department (Grant No. 202103070273), the Natural Science Foundation of Hunan Province (Grant No. 2021JJ70091), Changsha Science and Technology Plan Project (Grant No. kzd2001068), Funded Projected of The Affiliated Changsha Central Hospital, University of South China (Grant No. YNKY202211), and Hunan Province Science and Technology Innovation Key Project (Grant No. 2020SK1012).

## Conflict of interest

The authors declare that the research was conducted in the absence of any commercial or financial relationships that could be construed as a potential conflict of interest.

## Publisher's note

All claims expressed in this article are solely those of the authors and do not necessarily represent those of their affiliated organizations, or those of the publisher, the editors and the reviewers. Any product that may be evaluated in this article, or claim that may be made by its manufacturer, is not guaranteed or endorsed by the publisher.
