# Supplementary material for: A non-contrast computed tomography-based radiomics nomogram for the prediction of hematoma expansion in patients with deep ganglionic intracerebral hemorrhage
Source: Front Neurol. 2022 Oct 11;13:974183. doi: 10.3389/fneur.2022.974183 (PMC9592988; doi:10.3389/fneur.2022.974183)
Supplement: Supplementary file 1 [file Data_Sheet_1.docx]

Supplementary Material

Table S1 | Radiomics features used in the analysis

| Image type | Feature Class | No. of features |
| --- | --- | --- |
| Original image | First Order Statistics | 18 |
|  | Shape-based | 14 |
|  | Gray Level Cooccurence Matrix (GLCM) | 24 |
|  | Gray Level Run Length Matrix  (GLRLM) | 16 |
|  | Gray Level Size Zone Matrix  (GLSZM) | 16 |
|  | Neighbouring Gray Tone Difference Matrix (NGTDM) | 5 |
|  | Gray Level Dependence Matrix  (GLDM) | 14 |
| Gradient filtered image | First Order Statistics, GLCM, GLRLM, GLSZM, GLDM, NGTDM | 93 |
| Exponential filtered image | First Order Statistics, GLCM, GLRLM, GLSZM, GLDM, NGTDM | 93 |
| Logarithm filtered image | First Order Statistics, GLCM, GLRLM, GLSZM, GLDM, NGTDM | 93 |
| Local Binary Pattern (LBP) filterer image | First Order Statistics, GLCM, GLRLM, GLSZM, GLDM, NGTDM | 93 |
| Square filterer image | First Order Statistics, GLCM, GLRLM, GLSZM, GLDM, NGTDM | 93 |
| Square root filterer image | First Order Statistics, GLCM, GLRLM, GLSZM, GLDM, NGTDM | 93 |
| Wavelet-HHH filtered image | First Order Statistics, GLCM, GLRLM, GLSZM, GLDM, NGTDM | 93 |
| Wavelet-HHL filterer image | First Order Statistics, GLCM, GLRLM, GLSZM, GLDM, NGTDM | 93 |
| Wavelet-HLH filterer image | First Order Statistics, GLCM, GLRLM, GLSZM, GLDM, NGTDM | 93 |
| Wavelet-HLL filterer image | First Order Statistics, GLCM, GLRLM, GLSZM, GLDM, NGTDM | 93 |
| Wavelet-LHH filterer image | First Order Statistics, GLCM, GLRLM, GLSZM, GLDM, NGTDM | 93 |
| Wavelet-LHL filterer image | First Order Statistics, GLCM, GLRLM, GLSZM, GLDM, NGTDM | 93 |
| Wavelet-LLH filterer image | First Order Statistics, GLCM, GLRLM, GLSZM, GLDM, NGTDM | 93 |
| Wavelet-LLL filterer image | First Order Statistics, GLCM, GLRLM, GLSZM, GLDM, NGTDM | 93 |

Supplementary Table 2 | The definition and ICC value of selected radiomics features and radiological signs

| Feature Name | Image type | Feature Class | Description | ICC (95% CI) |
| --- | --- | --- | --- | --- |
| Gray Level Non Uniformity | Original | GLRLM | the similarity of gray-level intensity values in the image, where a lower value correlates with a greater similarity in intensity values. | 0.95 (0.91, 0.97)) |
| Short Run Emphasis | Wavelet-LLL filterer | GLRLM | a measure of the distribution of short run lengths, with a greater value indicative of shorter run lengths and more fine textural textures. | 0.97 (0.93,0.98) |
| Contrast | Wavelet-LLL filterer | NGTDM | a measure of the spatial intensity change. | 0.93 (0.88, 0.96) |
| Blend sign |  |  | an area of hematoma with low attenuation adjacent to an area of hematoma with high attenuation, with a density differing by at least 18 Hounsfield unit between the two areas | 0.85 (0.75, 0.91) |
| Hypodensity |  |  | a hypodense region within the area of hemorrhage with any shape or dimension, and that was not connected to the surrounding brain parenchyma | 0.88 (0.80, 0.93) |

GLRLM, Gray Level Run Length Matrix; NGTDM, Neighbouring Gray Tone Difference Matrix

Table S3 | Description of the Representative HE Predictive Model Based on the NCCT

| Predictive Model | Year | Variables in the Model | Definition of HE | Baseline CT Time | Follow-up CT Time | ICH location |
| --- | --- | --- | --- | --- | --- | --- |
| Model by Takeda (1) | 2013 | ICH volume, Hematoma heterogeneity, Elevated systolic blood pressure | >12.5ml or >33% | <6 hours | 24 hours from initial CT | Deep (basal ganglia) |
| BRAIN  (24-point Score) (2) | 2015 | Baseline ICH volume, Recurrent ICH, Anticoagulation with warfarin at onset, Intraventricular extension, Number of hours to baseline CT from symptom onset | >6ml or >33% | <6 hours | 24 hours from initial CT | N/A |
| HEP (3) | 2015 | Time from onset to baseline CT, History of dementia, Current smoking, Antiplatelet use, GCS score, and the Presence of subarachnoid hemorrhage on baseline scan | >6ml or >33% | <12 hours | 72 hours from initial CT | Lobar,  Deep,  Brainstem, Cerebellum |
| BAT (4) | 2018 | Blend sign, any Hypodensity, Time from onset to NCCT | >6ml or >33% | <6 hours | 24 hours from onset or earlier in case of clinical deterioration | supratentorial |
| 7-Point Score (5) | 2018 | Hours from onset to CT, Baseline ICH volume, Island sign, Blend sign, Swirl sign, Anticoagulant use or an INR >1.5, IVH extension | >6ml or >33% | <24 hours | 24 hours from initial CT | Deep (basal ganglia) |
| HEAVN (6) | 2018 | Heterogeneity, Peripheral edema, Anticoagulant use, ICH Volume >30 ml on initial CT, Niveau formation | >33% or >5mm of maximum diameter | N/A | 24 hours to 14 days after admission | Deep, loabr, posterior |
| NAG (7) | 2018 | Baseline NIHSS, Anticoagulants, Glucose | >6ml or >33% | <24 hours | 6 hours,24 hours or 7 days after admission or in case of neurological deterioration. | Deep, Lobar, Infratentorial, Intraventricular |
| 4-predictor model (8) | 2018 | Time from symptom onset to CT, baseline ICH volume, antiplatelet use, anticoagulant use | > 6ml | 0.5-24 hours | < 6 days | N/A |
| Model by Chen (9) | 2020 | Sex, GCS score, Time to baseline NCCT, R-score | >6ml or >33% | <6 hours | < 72 hours from onset | Lobar,  Deep,  Brainstem, Cerebellum |
| Model by Xu (10) | 2020 | Satellite sign number, R-score | >33% | <6 hours | 24 hours from initial CT | Basal ganglia, lobar, thalamus, brainstem |
| Model by Zhang (11) | 2022 | NLR, GCS score, Blend sign, Swirl sign, Hypodensities | >6ml or >33% | <6 hours | 24 hours from initial CT | Lobar,  Deep,  Brainstem, Cerebellum |

N/A, not applicable; GCS, Glasgow Coma Scale; NLR, neutrophil to lymphocyte ratio; R-score, radiomics score, HE, hematoma expansion, NCCT, non-contrast computed tomography

**Supplementary References**

1. Takeda R, Ogura T, Ooigawa H, Fushihara G, Yoshikawa S, Okada D, et al. A practical prediction model for early hematoma expansion in spontaneous deep ganglionic intracerebral hemorrhage. *Clin Neurol Neurosurg.* (2013) 115(7):1028-31. doi: 10.1016/j.clineuro.2012.10.016

2. Wang X, Arima H, Al-Shahi Salman R, Woodward M, Heeley E, Stapf C, et al. Clinical prediction algorithm (brain) to determine risk of hematoma growth in acute intracerebral hemorrhage. *Stroke.* (2015) 46(2):376-81. doi: 10.1161/strokeaha.114.006910

3. Yao X, Xu Y, Siwila-Sackman E, Wu B, Selim M. The HEP score: A nomogram-derived hematoma expansion prediction scale. *Neurocrit Care.* (2015) 23(2):179-87. doi: 10.1007/s12028-015-0147-4

4. Morotti A, Dowlatshahi D, Boulouis G, Al-Ajlan F, Demchuk AM, Aviv RI, et al. Predicting intracerebral hemorrhage expansion with noncontrast computed tomography: the BAT score. *Stroke* (2018) 49(5):1163-9. doi: 10.1161/strokeaha.117.020138

5. Huang Y, Zhang Q, Yang M. A reliable grading system for prediction of hematoma expansion in intracerebral hemorrhage in the basal ganglia. *Biosci Trends.* (2018) 12(2):193-200. doi: 10.5582/bst.2018.01061

6. Miyahara M, Noda R, Yamaguchi S, Tamai Y, Inoue M, Okamoto K, et al. New prediction score for hematoma expansion and neurological deterioration after spontaneous intracerebral hemorrhage: a hospital-based retrospective cohort study. *J Stroke Cerebrovasc Dis.* (2018) 27(9):2543-50. doi: 10.1016/j.jstrokecerebrovasdis.2018.05.018

7. Sakuta K, Sato T, Komatsu T, Sakai K, Terasawa Y, Mitsumura H, et al. The NAG scale: noble predictive scale for hematoma expansion in intracerebral hemorrhage. *J Stroke Cerebrovasc Dis.* (2018) 27(10):2606-12. doi: 10.1016/j.jstrokecerebrovasdis.2018.05.020

8. Al-Shahi Salman R, Frantzias J, Lee RJ, Lyden PD, Battey TWK, Ayres AM, et al. Absolute risk and predictors of the growth of acute spontaneous intracerebral haemorrhage: a systematic review and meta-analysis of individual patient data. *Lancet Neurol.* (2018) 17(10):885-94. doi: 10.1016/s1474-4422(18)30253-9

9. Chen Q, Zhu D, Liu J, Zhang M, Xu H, Xiang Y, et al. Clinical-radiomics nomogram for risk estimation of early hematoma expansion after acute intracerebral hemorrhage. *Acad Radiol.* (2021) 28(3):307-17. doi: 10.1016/j.acra.2020.02.021

10. Xu W, Ding Z, Shan Y, Chen W, Feng Z, Pang P, et al. A nomogram model of radiomics and satellite sign number as imaging predictor for intracranial hematoma expansion. *Front Neurosci.* (2020) 14:491. doi: 10.3389/fnins.2020.00491

11. Zhang X, Gao Q, Chen K, Wu Q, Chen B, Zeng S, et al. A predictive nomogram for intracerebral hematoma expansion based on non-contrast computed tomography and clinical features. *Neuroradiology*. (2022). doi: 10.1007/s00234-022-02899-9
